# Ultrafast photoresponse of vertically oriented TMD films probed in a vertical electrode configuration on Si chips†

**DOI:** 10.1039/d2na00313a

**Published:** 2022-07-05

**Authors:** Topias Järvinen, Seyed-Hossein Hosseini Shokouh, Sami Sainio, Olli Pitkänen, Krisztian Kordas

**Affiliations:** Microelectronics Research Unit, Faculty of Information Technology and Electrical Engineering, University of Oulu FI-90014 Oulu Finland topias.jarvinen@oulu.fi seyed.hosseinishokouh@oulu.fi; SLAC National Accelerator Laboratory, Stanford University Stanford CA 94025 USA

## Abstract

Integrated photodetectors based on transition metal dichalcogenides (TMDs) face the challenge of growing their high-quality crystals directly on chips or transferring them to the desired locations of components by applying multi-step processes. Herein, we show that vertically oriented polycrystalline thin films of MoS_2_ and WS_2_ grown by sulfurization of Mo and W sputtered on highly doped Si are robust solutions to achieve on-chip photodetectors with a sensitivity of up to 1 mA W^−1^ and an ultrafast response time in the sub-μs regime by simply probing the device in a vertical arrangement, *i.e.*, parallel to the basal planes of TMDs. These results are two orders of magnitude better than those measured earlier in lateral probing setups having both electrodes on top of vertically aligned polycrystalline TMD films. Accordingly, our study suggests that easy-to-grow vertically oriented polycrystalline thin film structures may be viable components in fast photodetectors as well as in imaging, sensing and telecommunication devices.

## Abstract 2

The probing geometry plays a decisive role in the photosensitivity and response time of vertically oriented TMD thin films.

## Graphical abstract

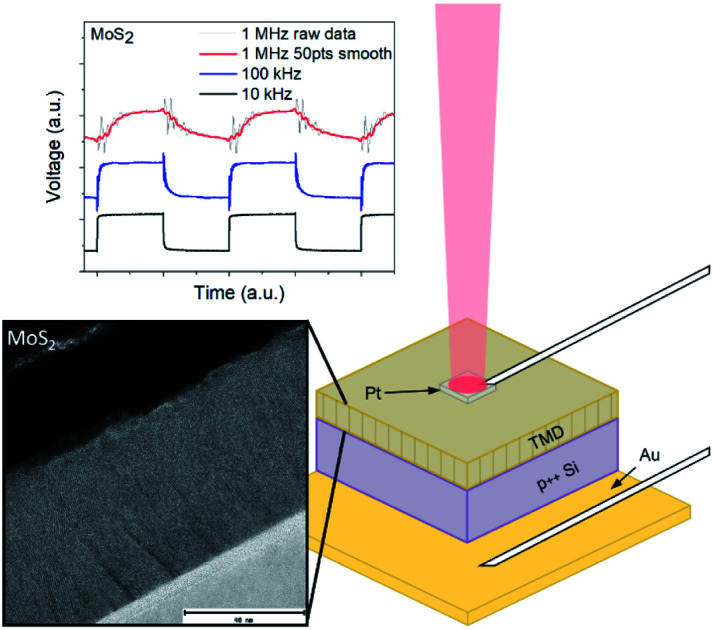

## Introduction

Among 2-dimensional (2D) materials, transition metal dichalcogenides (TMDs) such as disulfides and diselenides of Mo, W, V and Ti, show great potential for optoelectronic applications due to their thickness dependent intrinsic band gap,^1,2^ optical absorption in the visible spectrum,^3,4^ polarization dependency,^5–8^ high in-plane carrier mobility,^9,10^ and good chemical and thermal stability.^11,12^ While relatively weak, interlayer van der Waals forces allow easy exfoliation, single layers are mechanically strong which permits the fabrication of the TMD films into robust photodevices. As large-area growth of high-quality mono or few-layered TMDs, *e.g.*, on the wafer size, is not a mature technology yet,^13,14^ integrated optoelectronic devices of TMDs are mostly based on (i) small size single or few-layered flakes transferred from the growth substrate to the chip or (ii) large-area multilayers^15^ and bulk materials grown directly on chips.^16,17^

In most of the cases, lateral metal–semiconductor–metal electrode configurations are applied, which are optimal if the 2D crystal has lateral orientation. However, when the semiconducting layer is a thick polycrystalline film or it is thin but vertically aligned, the lateral electrode setup compromises the device response speed,^18^ which is an important property in applications such as in fast photodetectors, imaging and telecommunication.^19^ For this reason, thin films of TMDs with vertically oriented layers in the polycrystalline structure that are probed through vertical contacts have immense potential to compete with conventional devices based on planar single or few-layered structures. In addition, in vertically oriented devices, direct edge-contact of the 2D semiconductor with the metal electrodes provides a lower contact resistance^20^ than on the 2D planes of planar devices, and this further improves the device response speed, which is several orders of magnitude higher than in the lateral electrode arrangement (Table 1).

**Table 1:** Rise/decay time of vertically oriented TMD photodetector devices

Device	TMD synthesis method	Rise/decay time	Photoresponse (A/W)	Electrode geometry and material	Ref
MoS_2_ and WS_2_ on Si	Sulfurization	250/400 ns	1 × 10^−3^	Vertical, Pt	This work
MoS_2_ and WS_2_ on Si	Sulfurization	90–190 μs/150–470 μs (MoS_2)_	5.5 × 10^−6^ (MoS_2_)	Lateral, Pt	21
		40 μs/80–130 μs (WS_2_)	5.3 × 10^−6^ (WS_2_)		
PtSe_2_ on GaAs	Selenization	5.5/6.5 μs	262 × 10^−3^	Vertical, Ag (top)	22
MoS_2_/Cu_2_ZnS n–p junction on Si/SiO_2_	CVD	81/79 ms	141 × 10^−3^	Lateral, Au	23
MoS_2_ on Si	Sputtering	3/40 μs	∼300 × 10^−3^	Vertical, Ag	14
Few-layered HfS_2_ on Si/SiO_2_	CVD	24/24 ms	—	Lateral	24
Au-nanoparticle decorated MoS_2_ nanoflakes on Si/SiO_2_	CVD	<100/<100 ms	24 × 10^−3^	Lateral, Au	25
SnS_2_ nanoflakes on Si/SiO_2_	CVD	400 μs/560 μs	354.4	Lateral, Au/Cr	26
WS_2_/ZnO heterostructure on FTO/glass	Sputtering	0.8/2.2 ms	2.7	Vertical, ITO	27
MoSe_2_ on Si	Sputtering	270/350 ns	93–270 × 10^−3^	Vertical, graphene	19
MoS_2_ on Si/Ag	CVD	56/825 ns	908.2 × 10^−3^	Vertical, Ag	28

In this paper, the optoelectronic properties of vertically oriented and probed MoS_2_ and WS_2_ films grown on Si substrates are assessed, and compared to their counterparts having lateral electrode geometries we reported earlier.^21^ While the vertical setup is found to show good enhancement of photosensitivity, the response time of the devices improves significantly, by two orders of magnitude, compared to identical TMD films on Si/SiO_2_ chips but with lateral top contacts. Accordingly, our present study confirms that the synthesis of TMDs by sulfurization of metal thin films, and the subsequent application of sputtered semitransparent metal top electrodes offer a feasible approach to produce large-scale on-chip devices having ultrafast photodetection properties in the visible spectrum.

## Experimental

### Materials synthesis

Highly doped p^++^ Si wafers were cut into 10 × 10 mm^2^ square pieces using a laser (LPKF ProtoLaser U3, CMA Oulu). Mo or W films were then deposited on each wafer by sputtering (Torr International Physical Vapor Deposition (PVD) System, CMA Oulu, 2.4 × 10^−3^ torr Ar atmosphere, 155 W power, 1.2 Å s^−1^ deposition rate and 165 s total deposition time for Mo; 165 W power, 0.8–0.9 Å s^−1^ deposition rate and total deposition time of 235 s for W) with a nominal thickness of 20 nm (measured with Sigma instruments SQC-122c thin film deposition controller, CMA Oulu). The sulfurization process of the metal films follows the procedure described earlier. In short, Mo or W coated Si chips were inserted into a 2′′ tube furnace together with 1.00 g of sulfur powder (Sigma-Aldrich 215236) loaded in a ceramic boat. After evacuating with a dry rotary pump to <1 Torr pressure and purging the tube three times with N_2_ the temperature was raised to and kept at 800 °C for one hour, while keeping a constant 400 sccm flow of N_2_ during the synthesis.

## Materials characterization

The structure, orientation and composition of the films were analyzed using X-ray diffraction in the grazing incidence configuration (GIXRD, Co source, Rigaku SmartLab 9 kW) and Raman spectroscopy (Horiba Jobin-Yvon LabRAM HR800 with a laser wavelength of 488 nm). The GIXRD results were converted to equivalent Cu X-ray source patterns to allow for direct comparison to previous results.^29^ The data were plotted in OriginPro® 2020.

Cross-sections of the sulfurized films were prepared using a focused ion beam (FIB, FEI Helios DualBeam) and analyzed by transmission electron microscopy (TEM, JEOL JEM-2200FS EFTEM/STEM). In addition to the TEM/STEM imaging, energy-dispersive X-ray (EDX) spectroscopy and electron diffraction were performed to assess the chemical composition and crystal orientation.

To evaluate the chemistry, oxidation and potential contamination of the films, XPS and XAS spectra were collected at the Stanford Synchrotron Radiation Light source (SSRL) magnet beamline 8−2 that is equipped with a spherical grating monochromator, operated using 40 × 40 μm^2^ slits, resulting in a resolution of around 200 meV. The spot size at the interaction point was around 1 × 1 mm^2^, and the total flux was in the order of 10^10^ photons per s without any visible beam damage even for extended exposure. The X-ray energy for the C 1s, N 1s, and O 1s edges was scanned from 260 to 350, 380 to 430, and 520 to 560 eV, respectively. The XAS data in the TEY were collected using a drain current (amplified by using a Keithley picoammeter), and the AEY and XPS data were collected using a cylindrical mirror analyzer, operated with a pass energy of 200 eV and 50 eV, respectively. The incoming flux was recorded using a Ni grid with an Au sputtered film (called the i0). The data collected using the i0 follows the same procedure as the TEY measurements mentioned above. To normalize the XAS data, the slope of the linear background was varied within a small range to keep both the area and edge jump constant across the dataset. Before the peak fitting, the data was energy corrected and aligned to the C 1s sp^2^ π* at 285.2 eV. Furthermore, the C 1s, O 1s, and N 1s regions refer to the area normalized over the π and σ regions. More details of data normalization and energy correction can be found in ref.^30^ The XAS and XPS data analyses are carried out using OriginPro® 2020 software. Additional topology and structural analyses were performed by means of AFM and SEM/EDX on similar TMD films in our earlier study.^29^

### Device fabrication and photoconductivity measurements

The semitransparent top electrode on the chips was made by sputtering a thin Pt film with 8 nm thickness (Agar Auto Sputter Coater, Agar Scientific Ltd) through a laser-cut shadow mask (polycrystalline alumina wafer with 150 μm thickness having a 200 × 200 μm^2^ square pattern opening). The samples were then mounted onto a gold bottom electrode by applying silver paste.

The photoconductivity measurement setup is derived from the one used in our previous studies;^21,31^ a constant bias of 3 V is applied to the sample by using a sourcemeter (Keithley 2636A SYSTEM SourceMeter®) connected in series with a 1 MΩ load resistor. Three different lasers (Coherent® OBIS™ Laser System, TEM00 mode, with wavelengths of 661 nm, 552 nm, and 401 nm and 1/*e*^2^ beam diameters of 9 mm, 7 mm, and 8 mm, respectively) are modulated by using a signal generator (33220A, Agilent Technologies, Inc.) applying square waves with a duty cycle from 5% to 50% and frequency from 10 kHz up to 1 MHz depending on the laser wavelength. The signal generator is also used to trigger the oscilloscope (Agilent InfiniiVision DSO-X 3024A with 10 MΩ probe) connected in parallel with the load resistor. The acquired oscilloscope data are then analyzed using OriginPro® 2020 software to resolve the photocurrent, sensitivity, and response times.

## Results

### Structure and chemistry of TMD films

**Fig. 1:**
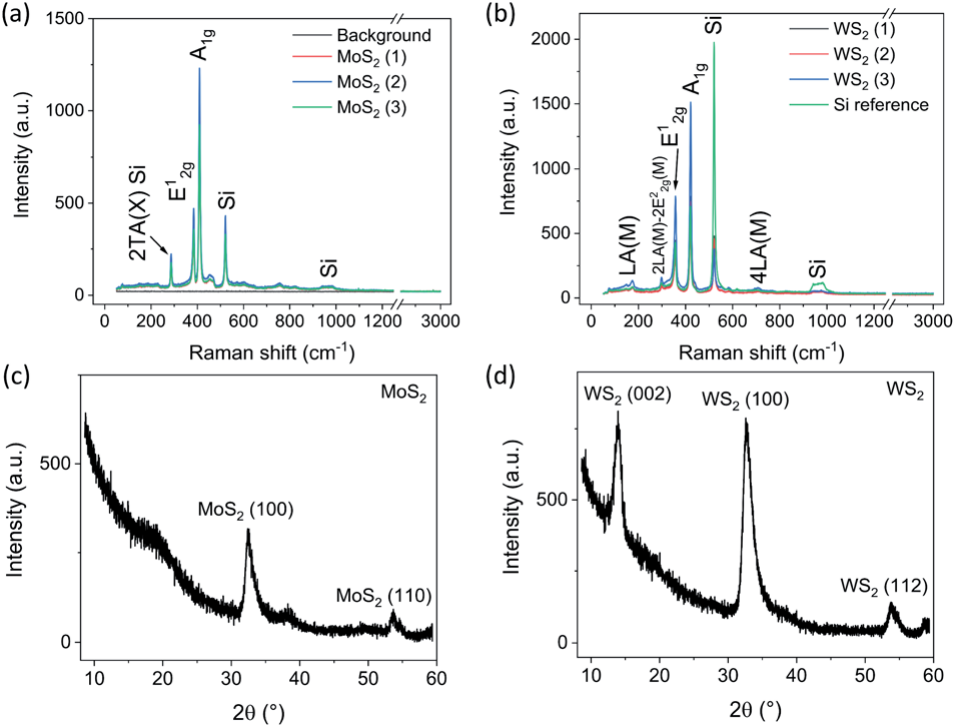
Raman spectra and XRD patterns of the TMD films. Raman spectra of the MoS_2_ (a) and WS_2_ (b) films labeled with corresponding phases. The irrelevant parts after 1200 cm^−1^ have been cut out. GIXRD patterns of MoS_2_ (c) and WS_2_ (d). Note that the 2*θ* angles of the original patterns obtained using a Co source are converted to the corresponding 2*θ* angles of the Cu source to enable direct comparison with the references.

The Raman spectra of the TMD films (Fig. 1(a) and (b) for MoS_2_ and WS_2_, respectively) indicate that the films are multilayered and crystalline.^29,32,33^ The two main peaks for MoS_2_ at 384 and 410 cm^−1^ resemble the in-plane (*E*^1^_2g_) and out-of-plane (*A*_1g_) vibrations, respectively.^34^ Likewise, WS_2_ (*E*^1^_2g_) and (*A*_1g_) peaks at 357 and 422 cm^−1^ agree with those in the literature and imply a multilayered structure.^35^ The longitudinal acoustic mode vibrations (LA) and harmonics (2LA and 4LA) appear for WS_2_ at 175, 298 and 707 cm^−1^, respectively.^33^ In addition to the peaks attributed to the TMD films, the measurements include a background noise level reference (Fig. 1(a)) as well as a pure silicon reference at 520 cm^−1^ (Fig. 1(b)).

X-ray diffraction patterns collected in the grazing incidence configuration (Fig. 1(c) and (d) for MoS_2_ and WS_2_, respectively) show reflections of MoS_2_ including (100) at 32.5° (PDF 75-1539) and (110) at 53.7° (PDF 73-1508). The lack of the (002) reflection implies a good vertical orientation of the 2D layers. In contrast, in the pattern of the WS_2_ film, the reflection of (002) at 13.9° (although having small relative intensity, PDF 08-0237) indicates that the grains of the 2D layer stacks are somewhat distorted.^21^

**Fig. 2:**
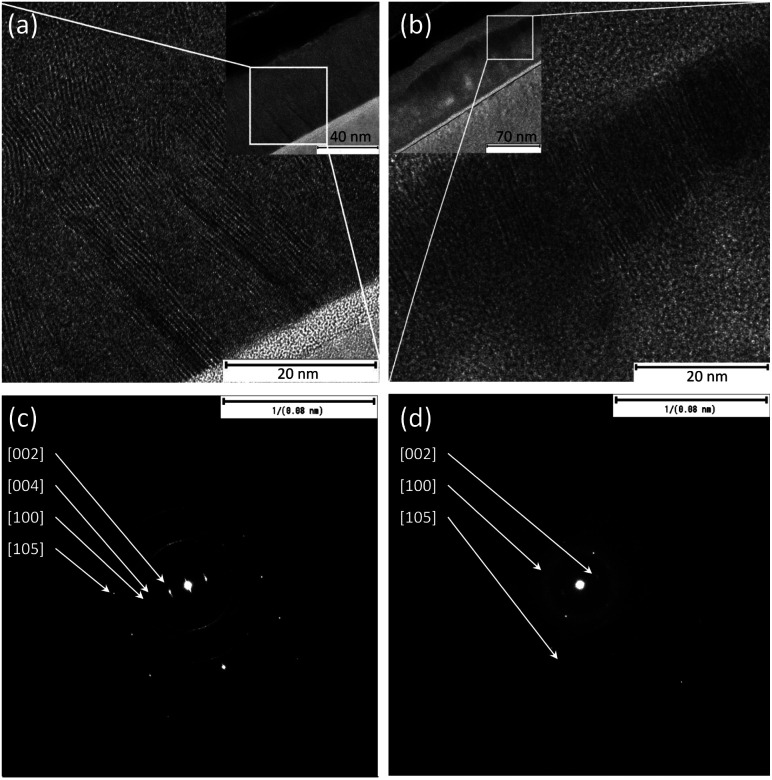
TEM images of cross-sections of (a) MoS_2_ and (b) WS_2_ grown on Si. Insets show complete films on Si substrates. Panels (c) and (d) display the corresponding electron diffraction patterns.

TEM analysis of the pure metal films reveals that the actual thicknesses are around 24–26 nm, which are slightly more than the originally specified nominal values of 20 nm. The sulfurized films show slightly varying thicknesses, approximately 55–61 nm for MoS_2_ and 85–110 nm for WS_2_. The latter is overall less uniform and has partially detached porous sections. Likewise, while the vertical orientation of MoS_2_ is clearly visible, WS_2_ shows somewhat misaligned grains of the crystalline layered structures (Fig. 2(a) and (b) for MoS_2_ and WS_2_, respectively). The same feature is visible in the electron diffraction patterns (Fig. 2(c) and (d) for MoS_2_ and WS_2_, respectively), in which for MoS_2_ we observe clear diffraction spots of the (002) plane, whereas for WS_2_ a diffraction ring is superposed on the spots due to the partial misalignment of the grains. The lattice spacings of the (002) plane measured from high-magnification TEM images are 6.5 ± 0.1 Å and 6.6 ± 0.1 Å for WS_2_ and MoS_2_, respectively. The corresponding values obtained from the electron diffraction patterns are somewhat lower (both 6.3 ± 0.1 Å).

EDX analysis of the film cross-sections gives a rough estimate of film stoichiometry (Table S1†). While the metal to sulfur ratio in MoS_2_ appears to be very close to the 1 : 2, WS_2_ contains a considerable amount of oxygen, which might be due to the chemical instability of WS_2_ at the crystal edges. Anyhow, despite the large amount of oxygen in the lattice, XRD and Raman analyses do not exhibit any phases of tungsten oxides.^29^

**Fig. 3:**
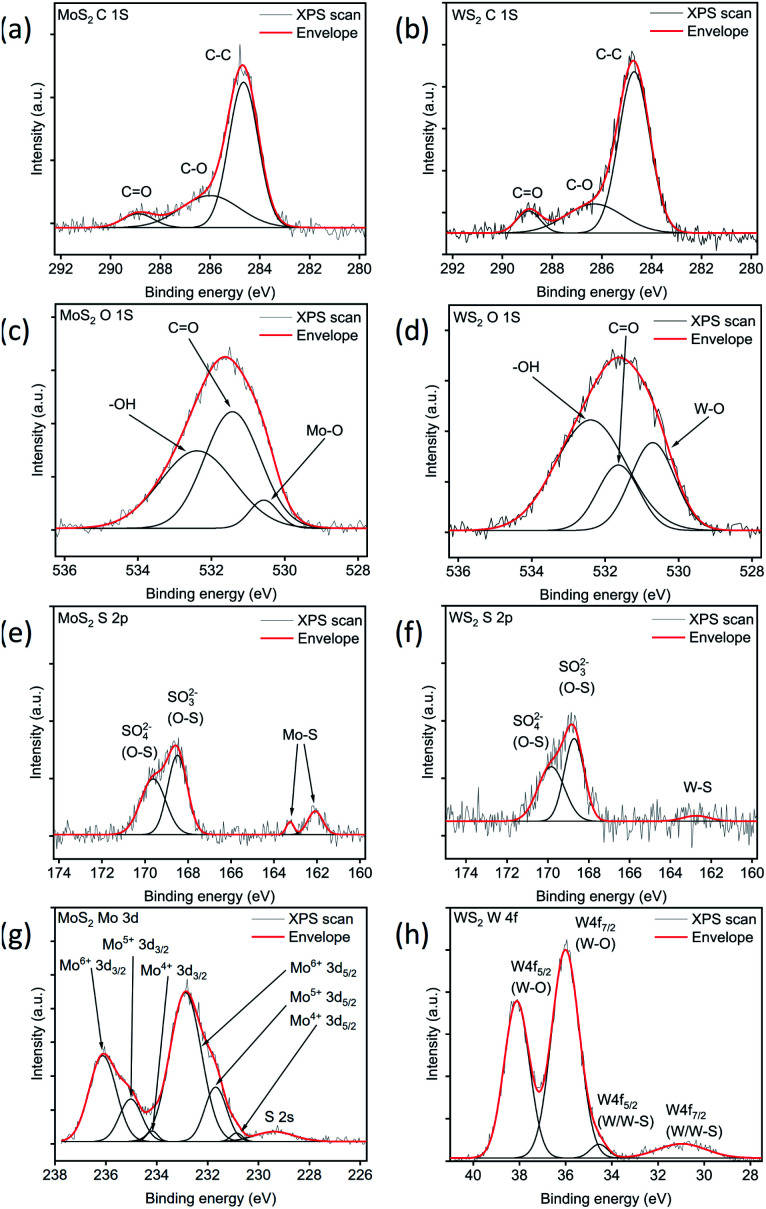
XPS analysis for carbon (a and b), oxygen (c and d), sulfur (e and f), molybdenum (g) and tungsten (h) bands.

Consequently, to have better insight into the chemistry of the thin films, we carried out further analyses using X-ray photoelectron and absorption edge spectroscopies (XPS and XAS, respectively). The XPS spectra of the MoS_2_ and WS_2_ samples show a carbon content which cannot be assigned to metal–carbon bonding indicating the presence of adsorbed carbon (Fig. 3(a) and (b)). In contrast, the O 1s peak reveals the oxygen–metal bonding in addition to adsorbed oxygen. For the MoS_2_ sample (Fig. 3(c)), the peak at 530.6 eV can be attributed to the Mo–O bond in addition to the adsorbed oxygen and hydroxyl/carboxyl groups at 531.4 and 532.4 eV, respectively.^36–38^ The resolved O 1s peak of WS_2_ (Fig. 3(d)) also indicates adsorbed oxygen (531.6 eV), hydroxyl/carboxyl groups (532.4 eV), and W–O bonds (530.7 eV).^39,40^

The Mo 3d and W 4f spectra also show the oxidation of metals. For instance, the Mo 3d spectrum (Fig. 3(g)) can be fitted with several peaks that correspond to the 4+, 5+ and 6 + oxidation states of molybdenum.^37,41–43^ Likewise, the W 4f spectrum (Fig. 3(h)) reveals the W–O bonds at 36.0 and 38.1 eV in addition to the W 4f_7/2_ (31.0 eV) and W 4f_5/2_ (34.6 eV) doublets of the W–S bond.^38,39,44–47^

While resolving sulfur-oxygen bonding from O 1s spectra is impossible, the S 2p spectra reveal the sulfur-oxygen bonding. For MoS_2_, the peaks (Fig. 3(e)) can be resolved as a doublet with components at 162.0 and 163.3 eV of S 2p_3/2_ and S 2p_1/2_ of the Mo–S bond,^36,48^ while the other two peaks at 168.5 and 169.6 eV imply the presence of sulfite/sulfate (SO_3_^2−^/SO_4_^2−^) traces.^37,49^ In the case of WS_2_, the S 2p spectrum (Fig. 3(f)) also indicates the presence of SO_3_^2−^ (168.7 eV) and SO_4_^2−^ (169.8 eV)^38,49^ in addition to the W–S bond (162.7 eV). The peak assignments are listed in the ESI (Table S2†).

To further evaluate the contamination and oxidation of the thin films, the C, and O K edge absorption spectra of the WS_2_ and MoS_2_ samples were collected at an incidence angle of 55° (magic angle) along with the Mo M_2,3_ edges at grazing and normal incidence angles of 20°, 55° and 90°. The Auger electron yield (AEY) and total electron yield (TEY) C1s spectra with a missing exciton signature at 291.65 eV, and clear σ* resonance above circa 292 eV indicate the lack of long-range order in the sp^2^ network (Fig. S1†).^30,50^ Moreover, the absence of metal-carbon bonding features, same as in the XPS data, implies the presence of carbon as a contaminant (organic compound) on the MoS_2_ and WS_2_ thin film surfaces. Especially for the MoS_2_ sample, Mo M-edge spectra confirm the absence of metal-carbon bonding, for there is no peak *ca.* 395.4 eV.^51^

On the other hand, the O K edge absorption spectra, containing oxygen–metal bonding features, reveal the partial surface oxidation of the MoS_2_ and WS_2_ samples. In agreement with the EDX data and O K edge absorption spectra, the Mo M-edge spectra show an oxidation state of +6^52^ (instead of +4) for the Mo atom, further supporting the surface oxidation of the MoS_2_ sample. The comprehensive data analysis and peak assignment are provided in the ESI (Fig. S1–S3 and Tables S3, S4†).

### Photoresponse of vertically aligned TMD films measured with vertical probing

**Fig. 4:**
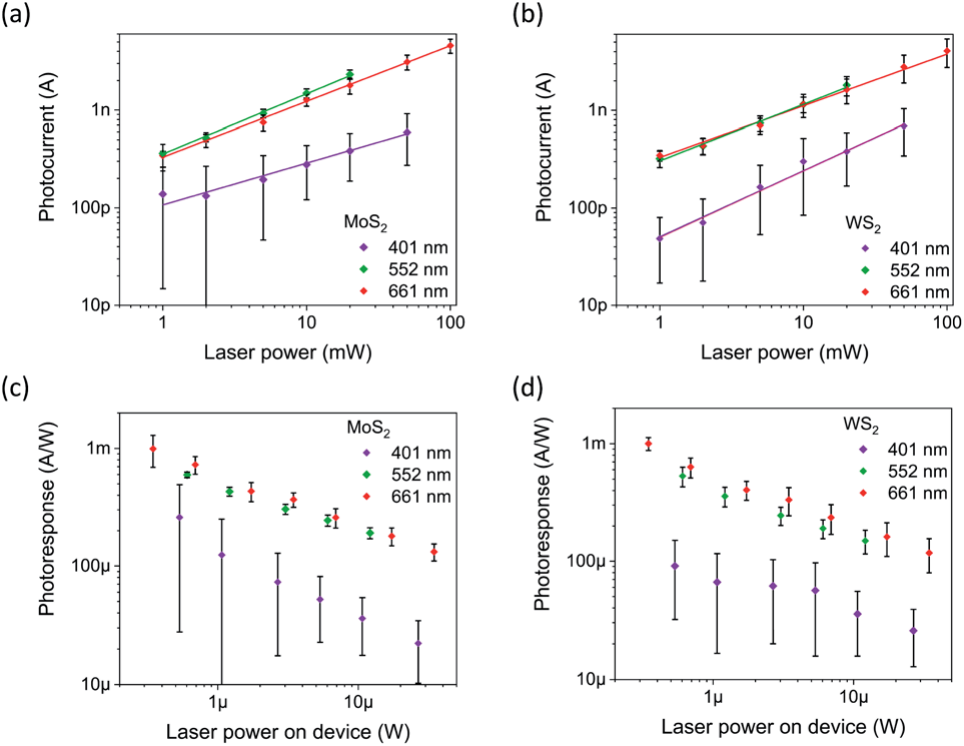
Photocurrents (a and b) and photoresponses (c and d) for MoS_2_ and WS_2_ photodevices, respectively.

Despite the impurities and oxidation especially in the case of WS_2_, both films show excellent response to illumination with laser pulses in the visible range, Fig. 4. The measured photocurrents are strongly dependent on the applied laser power at each wavelength and can be fitted well using a simple power function (*y* = *ax*^*b*^). The exponents fitted from the calculated mean values (Table 2) show variance between 0.43 and 0.68, which correspond to mid-gap states in the semiconductor, scattering events and possibly even saturable absorption,^53,54^ and are similar to reported values for 2D TMD materials such as single-layered WS_2_ (0.4),^55^ as well as multilayered WS_2_ (0.24–0.38) and MoS_2_ (0.35–0.67).^21,56^ The measurements at 401 nm introduced persistent photoconductivity, which could partly be attributed to traps due to the oxidation of the film. This was addressed by reducing the duty cycle from 50% to 5% which also lowered the photocurrent compared to the 552 and 661 nm measurements performed at 50%. Due to different duty cycles, wavelength dependency of the photoresponses cannot be accurately determined.

**Table 2:** Photocurrent power fit exponent values for MoS_2_ and WS_2_ at different wavelengths

Wavelength (nm)	MoS_2_	WS_2_
401	0.43	0.68
552	0.62	0.58
661	0.57	0.53

In order to make an accurate assessment of the photosensitivity, the incident laser beam power on the illuminated square-shaped electrode is calculated by integrating the Gaussian beam over the electrode area, also considering the incidence angle of 70°. In addition, reflectance and absorbance of the 8 nm thick Pt top electrode are accounted for. Furthermore, as the vertical TMD structures have considerably low conductivity in the lateral direction, we assumed that photo-carriers generated outside the electrode covered film have negligible contribution to the photocurrent. The as-obtained maximum photosensitivity values are 0.99 mA W^−1^ for MoS_2_ and 1.00 mA W^−1^ for WS_2_, which are over two orders of magnitude higher than those measured with horizontal electrodes in our previous study.^21^ This difference can partially account for the differences in the electrode areas but even the raw photocurrent values are considerably higher.

**Fig. 5:**
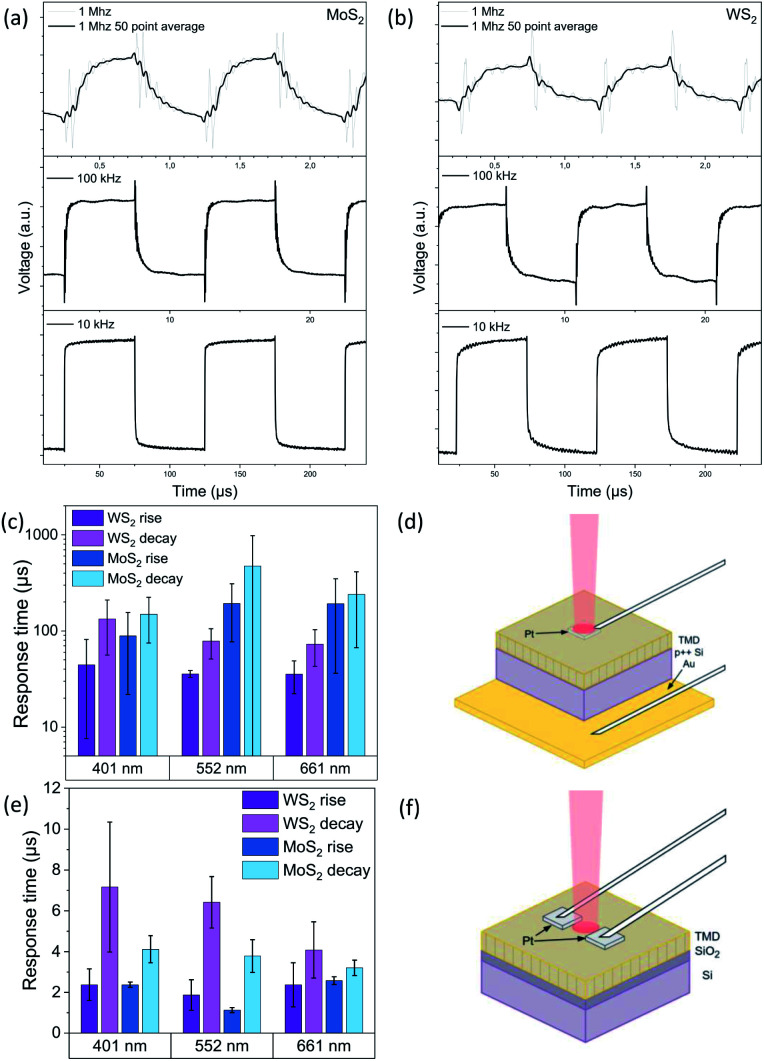
Plotted photoresponses for a vertical electrode configuration at 10 kHz, 100 kHz and 1 MHz for MoS_2_ (a) and WS_2_ (b). Response time statistics (c) and measurement setup (d) for the vertical electrode configuration (this study). Respective statistics (e) and setup (f) for lateral electrodes reported in our previous study.^17^

The most remarkable difference between the electrode configurations is undisputed, when analyzing the response times. Statistics were collected from the 10 kHz signals at 10 mW for all wavelengths (Fig. 5(c)). Examples for raw waveform signals used for statistics are presented in the ESI (Fig. S4†). As the timescale resolution did not provide accurate exponential fits at the 10 kHz signal, the time constants for the rise and decay transients were calculated from the corresponding 10% and 90% values of the photocurrents providing time constants in the range of a few microseconds. However, when analyzing the waveforms at higher frequencies such as at 1 MHz (Fig. 5(a) and (b) for MoS_2_ and WS_2_, respectively), it becomes clear that the actual rise and fall times are well below microseconds for the vertical electrode configuration (approx. 250 ns and 400 ns, respectively). The 50 point averaged signals were also fitted with double exponentials to determine the time constants more precisely (Table S5†). The noise introduced by the measurement setup as well as the 50 kHz upper limit for modulating the 552 nm laser prevent a more comprehensive set of measurements at higher frequencies. The vertical electrode configuration is presented in Fig. 5(d), while response time statistics and the lateral electrode configuration collected and adapted from our previous study are shown in Fig. 5(e) and (f), respectively.^21^

The striking difference between the photocurrents and photoresponse speeds measured between the lateral and vertical contacts on vertically aligned 2D TMD crystals stems from a number of events. The crystal anisotropy and the presence of tunneling barriers at the van der Waals interlayers result in direction dependent carrier mobility and electron–phonon interactions which favor in-plane transport.^57^ The presence of long-life interlayer excitons^58^ that form between electron and hole adjacent layers induces local polarization and may influence carrier generation and transport. Furthermore, although it is known that point defects^59^ in the 2D layers can enhance interlayer transport (within the grains), our films are polycrystalline, *i.e.*, the vertically oriented crystals share boundaries complicating the picture even further. As such grain boundaries act as scattering centers, enhance the recombination of photogenerated carriers, and also introduce trap states and barriers, transport^60^ in the horizontal direction across vertically oriented grains is expected to be significantly limited compared to transport in the vertical direction, in qualitative agreement with our experimental data. It is worth noting that due to the polycrystalline nature of the vertically oriented grains in the films, light polarization dependency unlike in laterally oriented, anisotropic TMD films and flakes^5–8^ is not expected.

## Conclusions

Photocurrent measurements with visible laser pulses on vertically oriented and probed MoS_2_ and WS_2_ films indicate a significant improvement in both photosensitivity and response times with reference to those measured with horizontal probe setups. The photosensitivity was found to increase by an order of magnitude, whereas the photocurrent rise and decay times decreased by two orders of magnitude to less than 1 μs because of the enabled in-plane carrier transport and thus, reduced scattering/recombination of photogenerated carriers. The response times could be further enhanced by limiting capacitive effects between the substrate and top electrode as well as minimizing the resistance at contact interfaces between the TMD film and electrodes. Accordingly, our study suggests that in photodetector applications, vertically oriented polycrystalline TMD films shall be probed across the film. Furthermore, as the size of the device is only limited by the dimensions of the semitransparent top electrode, our study suggests the possibility of fabricating sensor arrays with a high spatial resolution for advanced photodetector applications. The direct on-chip synthesis method providing large-scale films brings opportunities for scale-up production and commercial applications, provided that the film homogeneity and packaging are optimized. Furthermore, as micropatterned TMD films can be produced by a variety of lithography based methods,^61–63^ optimization of on-chip vertical in-plane device performance by adjusting the geometry is enabled and expected in subsequent studies.

## Author contributions

Conceptualization – T. Järvinen, S.-H. H. Shokouh, K. Kordas. Investigation – T. Järvinen, S.-H. H. Shokouh, O. Pitkänen, S. Sainio. Supervision – K. Kordas. Visualization – T. Järvinen, S.-H. H. Shokouh. Writing (original draft) – T. Järvinen, S.-H. H. Shokouh. Writing (review & editing) – T. Järvinen, S.-H. H. Shokouh, K. Kordas.

## Conflicts of interest

There are no conflicts to declare.

## Supplementary Material

NA-004-D2NA00313A-s001
