# Supplementary material for: Ultrafast photoresponse of vertically oriented TMD films probed in a vertical electrode configuration on Si chips
Source: Nanoscale Adv. 2022 Jul 5;4(15):3243–9. doi: 10.1039/d2na00313a (PMC9417830; doi:10.1039/d2na00313a)
Supplement: NA-004-D2NA00313A-s001 [file NA-004-D2NA00313A-s001.pdf]

Electronic Supplementary Material (ESI) for Nanoscale Advances.
This journal is © The Royal Society of Chemistry 2022

Supporting Information

Ultrafast photoresponse of vertically oriented TMD films probed in a vertical electrode configuration on Si chips

Topias Järvinen, Seyed-Hossein Hosseini Shokouh*, Sami Sainio, Olli Pitkänen, Krisztian Kordas*

Table S1. Collected element stoichiometry from EDX analysis on the MoS_2 and WS_2 films.

Element	O	Si	S	W	Mo	M:S ratio
MoS_2	5.7	1.4	60.9	-	31.1	1:1.93
WS_2	69.9	1.9	5.5	22.2	-	1:0.25

Table S2. XPS peak assignment.

Sample	Peak	Binding energy (eV)	Assignment
MoS_2	C 1s	284.7	C–C
		286.0	C–O
		288.9	C=O
	O 1s	530.6	Mo–O
		531.4	C=O
		532.4	–OH
	S 2p	162.0	Mo–S
		163.3	Mo–S
		168.5	SO_3^{2-}
		169.6	SO_4^{2-}
	Mo 3d	229.4	Mo–S
		230.9/234.2	Mo^{4+}
		231.7/235.0	Mo^{5+}
		232.9/236.1	Mo^{6+}
WS_2	C 1s	284.7	C–C
		286.3	C–O
		289.0	C=O
	O 1s	530.7	W–O
		531.6	C=O
		532.4	–OH
	S 2p	162.7	W–S
		168.7	SO_3^{2-}
		169.8	SO_4^{2-}
	W 4f	31.0/34.6	W–S
		36.0/38.1	W–O

AEY and TEY analysis

Figure S1 A and B show the AEY and TEY spectra of C1s edge for WS_2 sample. On the TEY spectra, the peaks at 285.3 and 288.65 eV are assigned to the transitions of C 1s electrons to the p* molecular orbital of C=C in the sp^2 network and C=O in the carboxyl groups (COOH), respectively.[1,2] The σ* region is typically characterized by features ranging from 291 eV for single bonds carbon to 310 eV for triple bonds.[3,4] Thus, the peak at 294.25 eV matches the σ* excitation of C-O bonding apparently in the acrylic acid configuration.[5] And the broad peak at 301.86 eV can originate from the sigma features of C=O and C=C in the carboxyl and acrylic acid, respectively.[1,5] The sharp peak at 308.26 eV in the AEY spectra, in contrast to the relatively broad features of the σ region, is likely arising from Si2p 3rd order which is visible only in the WS_2 sample as some Si is exposed though the film.[1]

For the MoS_2, the spectra follow the same trend, Fig. S1 C and D. At 285.3 and 288.65 eV, the absorption edges correspond to the transitions of C1s electrons to the p* C=C molecular orbital in sp^2 bonding and C=O molecular orbital in carboxyl groups, respectively.[1,2] The peak at 293.35 eV can be assigned to σ* excitation for carbon single bonded to oxygen (C-O), probably in Methyl formate configuration (~ 293.7 eV),[5] while the peak at 297.35 eV corresponds to σ* resonance of single-bonded carbon and oxygen either in C–OH or C–O–C configuration.[2] And the peak at 299.95 eV is very close to the σ *(C=C) resonance in propenol that is around 300 eV.[2,5]

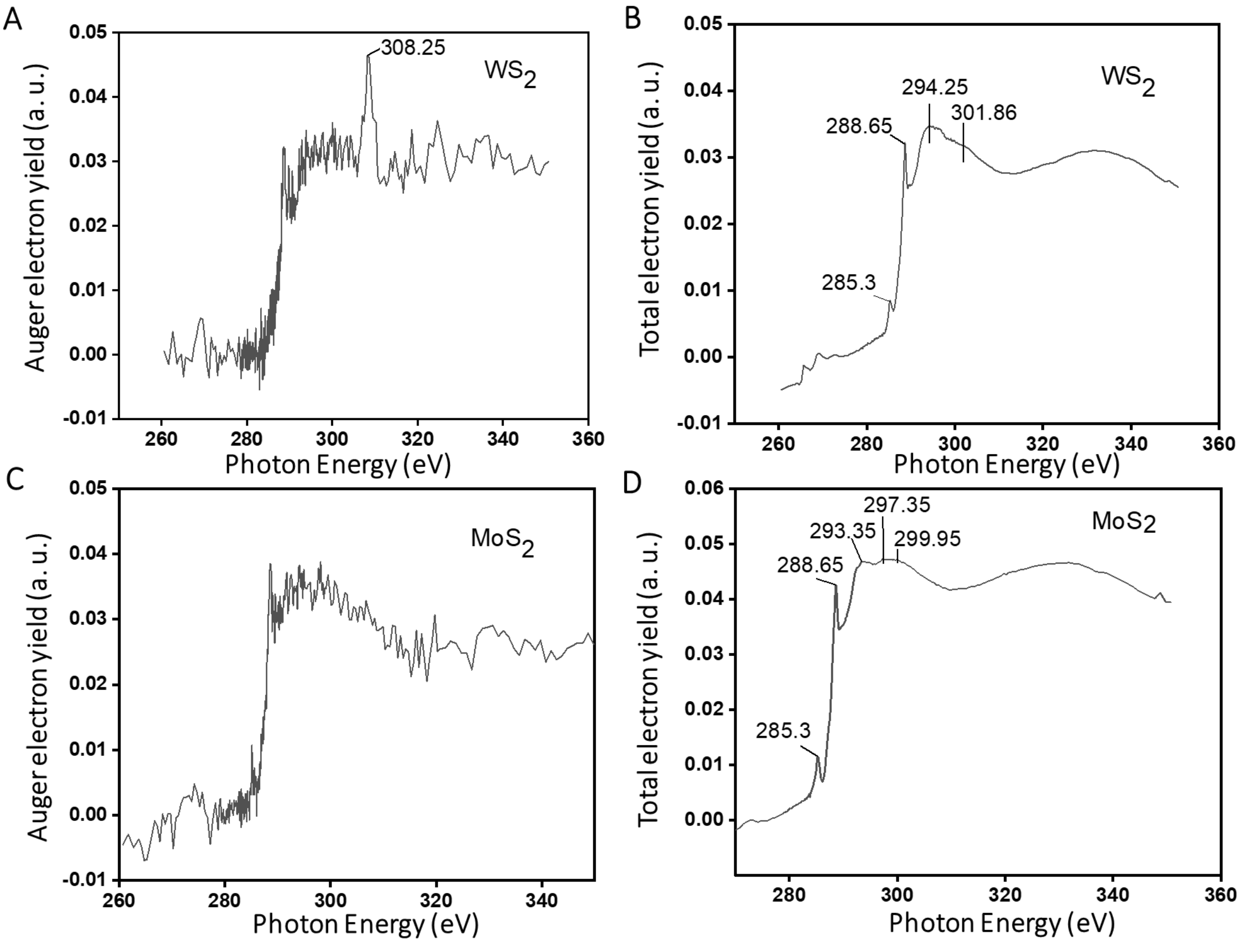

Figure S1. C 1s edge absorption spectra for WS_2 in (A) AEY and (B) TEY mode and for MoS_2 in (C) AEY and (D) TEY mode.

The O K edge absorption spectra contain features of oxygen-carbon bonding as a surface contaminant; however, in contrast to the carbon, the spectra show metal-oxygen bonding as well, **Figure S2**. For the WS_2 sample, the pre-edge at 530.25 eV represents the t_{2g} band formed by W 5d and O 2p orbitals, Fig. S2 A and B, while the shoulder at 531.6 eV is related to the anisotropy of this band.[2,6,7] It is worth mentioning that the π* resonances from double-bonded oxygen to carbon (carbonyl, C=O) are also expected around 530.8 and 531.8 eV.[2] The e_g peak (formed by W 5d and O 2p hybridization) cannot be well resolved here due to the overlap of the wide feature at 536.75 eV. The relatively lower intensity of t_{2g} peak compared to e_g, in contrast to previous studies, might be attributed to the modification of the chemical environment of W–O bonding (e.g., the W reduction in WO3 to the W3O phase transition as reported in our previous study [3,7,8]) or the association of the broad peak at 536.75 eV.

The relatively wide feature at 542.04 eV corresponds to the W 6sp and O 2p hybridization, leading to energy separation of $D(E_{sp} – E_{d1})$ around 11.79 eV that is in good agreement with the previous

reports.[3,9] Finally, with the peak at 564.65eV, corresponding to the scattering resonances at the nearest atoms, the TEY spectrum resembles that of the a-WO_3 spectra reported in the literature.[3,6] The O K edge absorption for the MoS_2 sample follows the same analogy, Figure S2 C and D, resembling the a-MoO_3 spectra.[3,9] The peaks at 529.75 eV and 531.9 eV correspond to the t_{2g} band formed by Mo 4d and O 2p hybridization.[6] While the wide feature at 537.15 is probably overlap of peaks resulting from the e_g orbital, and s* resonance of single-bonded oxygen in either C-O-C or C-O-H configurations.[2,3,6] The peaks at 542.85 and 562.75eV, same as the WS_2 sample, correspond to the Mo 5sp and O 2p hybridization and the scattering resonances at the nearest atoms, respectively.[6]

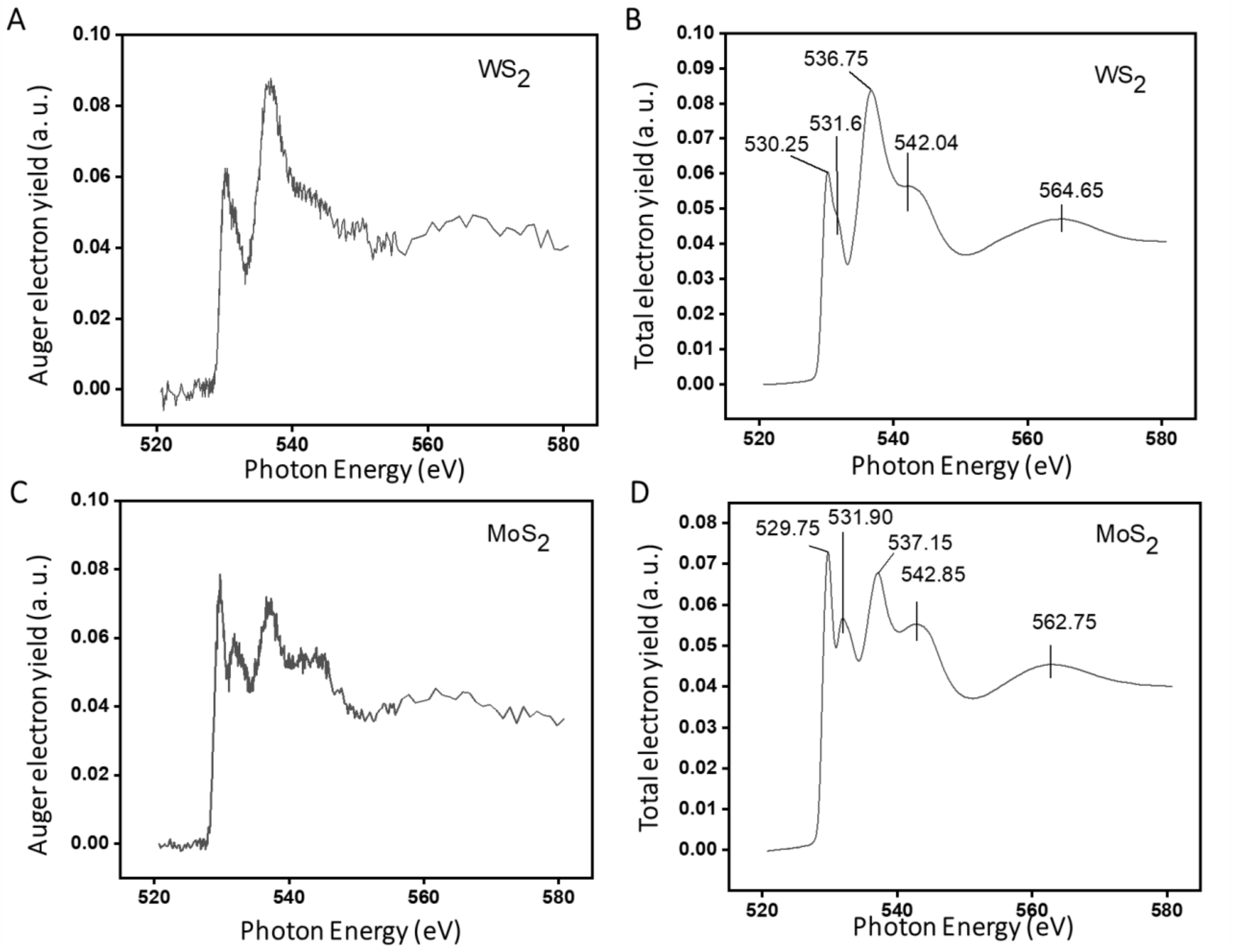

Figure S2. O 1s edge absorption spectra for WS_2 in (A) AEY and (B) TEY mode and for MoS_2 in (C) AEY and (D) TEY mode.

Finally, the molybdenum oxidation state was calculated, via a linear relationship, from the energy positions of Mo M-edge features. The polarization-dependent spectra of Mo $M_{2,3}$ were collected with a grazing incidence angle of 20˚, 55˚, and 90˚, **Figure S3**. For the AEY spectra, limited to a depth profile of 1 nm, the peaks originating from the N atom, appearing in the same spectral range,

are probably dominant, while for TEY spectra, with a depth profile of 10 nm, the Mo $M_{2,3}$ absorption edges are prominent.

The Mo M_3 edge absorption, at 398.5 and 400.5 eV [3], implies the partial oxidation of the MoS_2 surface with the oxidation state of +6 (instead of +4) for Mo.[10]

The other peaks at 415.5 and 418.1 eV are ascribed to the Mo M_2 edge, while the broad and polarization-dependent peak at 406.9 eV can be assigned to the N K edge absorption.[11,12]

For clarity and comparison, the peak assignments are summarized in **Table S3** and **S4**.

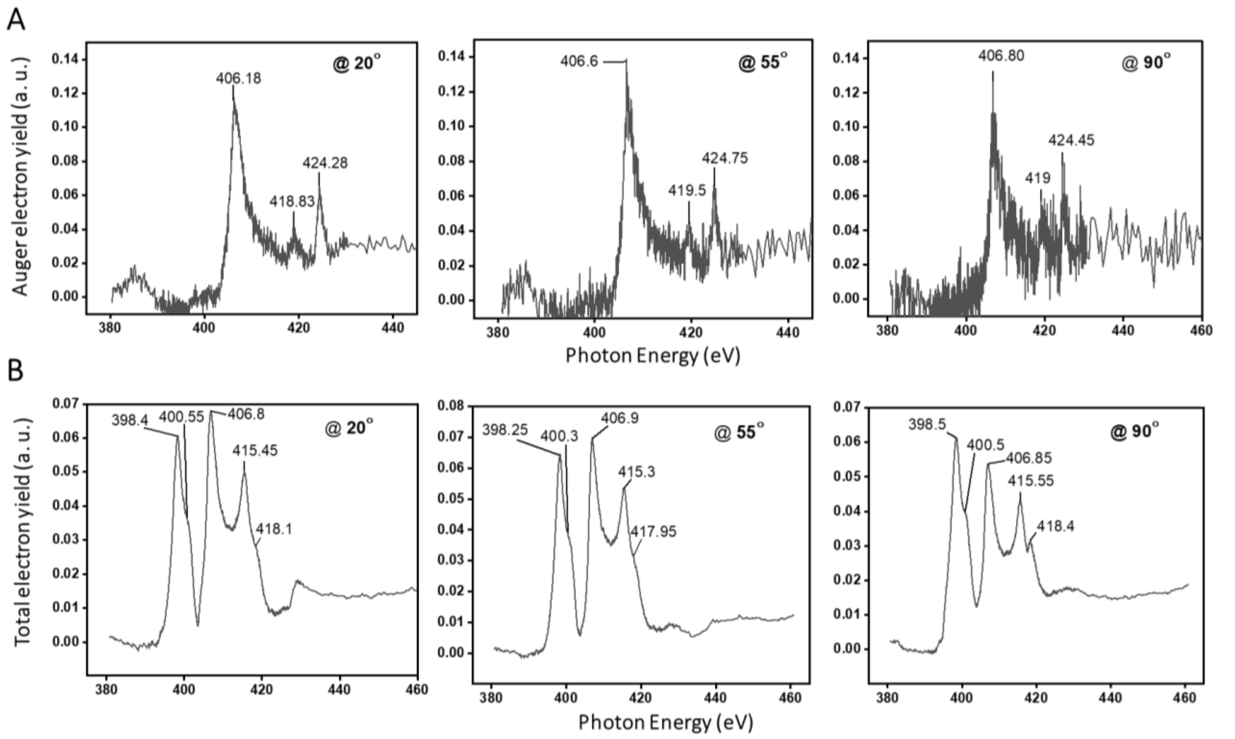

Figure S3. (A) AEY and (B) TEY spectra of Mo $M_{2,3}$ edge absorption at incidence angle of 20˚, 55˚, and 90˚**.**

Table S3. C 1s and O 1s peak assignment at incidence angle of 55˚ for MoS_2 and WS_2 samples.

Sample	Peak	Spectra	Photon energy (eV)	Assignment (transition)	Ref.
**WS_2**	C 1s	TEY	285.3	π * (C=C)	[1,2]
		TEY	288.65	π*(C=O) resonances in carboxyl groups (COOH)	[1,2]
		TEY	294.25	σ* (C-O)	[5]
		TEY	301.86	σ* (C=C or C=O)	[1,5]
		AEY	308.26	Si 2p	[1]
	O 1s	TEY	530.25	t_{2g} (hybridized orbitals of W 5d + O 2p)	[2,6,7]
		TEY	531.6	t_{2g} (hybridized orbitals of W 5d + O 2p)	[2,6,7]
		TEY	536.75	e_g (hybridized orbitals of W 5d + O 2p) or σ* (C−OH / C-O-C)	[2,3]
		TEY	542.04	Hybridized orbitals of (W 6sp+ O 2p)	[3,9]
		TEY	564.65	Scattering resonances at the nearest atoms	[3,6]
**MoS_2**	C 1s	TEY	285.25	π * (C=C)	[1,2]
		TEY	288.65	π*(C=O) resonances in carboxyl groups (COOH)	[1,2]
		TEY	293.35	σ* (C-O)	[5]
		TEY	297.35	σ* resonance attributed to single-bonded oxygen e.g., C–OH or C–O–C	[2]
		TEY	299.95	σ *(C=C)	[2,5]
	O 1s	TEY	529.75	t_{2g} (hybridized orbitals of Mo 4d + O 2p)	[6]
		TEY	531.9	t_{2g} (hybridized orbitals of Mo 4d + O 2p)	[6]
		TEY	537.15	e_g (hybridized orbitals of Mo 4d + O 2p) or σ* (C−OH / C-O-C)	[2,6]
		TEY	542.85	Hybridized orbitals of (Mo 5sp+ O 2p)	[3,9]
		TEY	562.75	Scattering resonances at the nearest atoms	[6]

Table S4. Mo M_{III} and M_{II} edge absorption at incidence angle of 20˚, 55˚, and 90˚.

Photon energy (eV)			Assignment (transition)	Ref.
**20°**	**55°**	**90°**		
398.4	398.25	398.5	$M_{III}(t_{2g})$	[3,10]
400.55	400.3	400.5	M_{III} (e_g)	[3]
406.8	406.9	406.85	N-O/H/Si	[11,12]
415.45	415.3	415.55	M_{II} (t_{2g})	[3]
418.1	417.95	418.4	M_{II} (e_g)	[3]

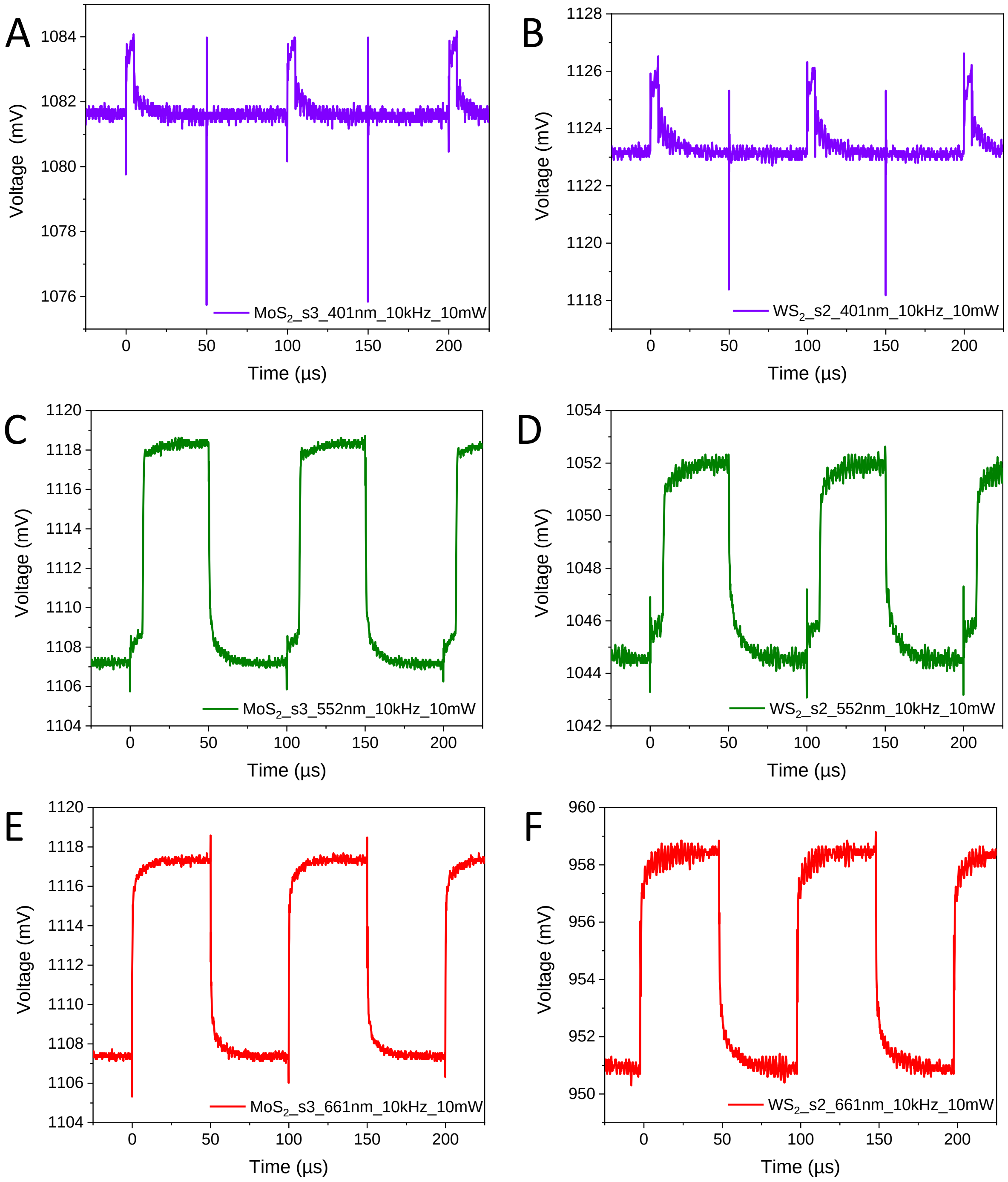

Figure S4. Example of raw waveforms used in photoresponse statistics calculations. Note that the 405 nm was pulsed at 5% duty cycle (A for MoS_2, B for WS_2) whereas 552 nm and 661nm were pulsed at 50 % duty cycle. In case of 552 nm pulses a slight delay in full laser power delivery can be seen in the rising edge.

Table S5. Collected response times with both 10-90% and double exponential fittings from 50-point averaged 1 MHz signals at 661 nm.

Sample	Rise time 10-90% (ns)	Decay time 90-10 % (ns)	Rise time with double exponential fitting t_1 / t_2 (ns)	Decay time with double exponential fitting t_1 / t_2 (ns)
**MoS_2**	250	370	11 / 86	49 / 113
**WS_2**	330	400	78 / 80	115 / 115

References

(1) Sainio, S.; Wester, N.; Aarva, A.; Titus, C. J.; Nordlund, D.; Kauppinen, E. I.; Leppänen, E.; Palomäki, T.; Koehne, J. E.; Pitkänen, O.; Kordas, K.; Kim, M.; Lipsanen, H.; Mozetič, M.; Caro, M. A.; Meyyappan, M.; Koskinen, J.; Laurila, T. Trends in Carbon, Oxygen, and Nitrogen Core in the X-Ray Absorption Spectroscopy of Carbon Nanomaterials: A Guide for the Perplexed. *J. Phys. Chem. C* **2021**, *125* (1), 973–988. https://doi.org/10.1021/acs.jpcc.0c08597.

(2) Gandhiraman, R. P.; Nordlund, D.; Javier, C.; Koehne, J. E.; Chen, B.; Meyyappan, M. X-Ray Absorption Study of Graphene Oxide and Transition Metal Oxide Nanocomposites. *J. Phys. Chem. C* **2014**, *118* (32), 18706–18712. https://doi.org/10.1021/jp503941t.

(3) J. G. Chen. NEXAFS Investigations of Transition Metal Oxides, Nitrides, Carbides, Sulfides and Other Interstitial Compounds. *Surf. Sci. Rep.* **1997**, *30* (1), 1–152. https://doi.org/10.1016/S0167-5729(97)00011-3.

(4) Stöhr, J. *NEXAFS Spectroscopy*; Springer Series in Surface Sciences; Springer-Verlag: Berlin Heidelberg, 1992. https://doi.org/10.1007/978-3-662-02853-7.

(5) Ishii, I.; Hitchcook, A. P. The Oscillator Strengths for C1s and O1s Excitation of Some Saturated and Unsaturated Organic Alcohols, Acids and Esters. *J. Electron Spectrosc. Relat. Phenom.* **1988**, *46* (1), 55–84. https://doi.org/10.1016/0368-2048(88)80005-7.

(6) Purans, J.; Kuzmin, A.; Parent, Ph.; Laffon, C. X-Ray Absorption Study of the Electronic Structure of Tungsten and Molybdenum Oxides on the O K-Edge. *Electrochimica Acta* **2001**, *46* (13), 1973–1976. https://doi.org/10.1016/S0013-4686(01)00370-X.

(7) Hu, A.; Jiang, Z.; Kuai, C.; McGuigan, S.; Nordlund, D.; Liu, Y.; Lin, F. Uncovering Phase Transformation, Morphological Evolution, and Nanoscale Color Heterogeneity in Tungsten Oxide Electrochromic Materials. *J. Mater. Chem. A* **2020**, *8* (38), 20000–20010. https://doi.org/10.1039/D0TA06612E.

(8) Järvinen, T.; Lorite, G. S.; Peräntie, J.; Toth, G.; Saarakkala, S.; Virtanen, V. K.; Kordas, K. WS_2 and MoS_2 Thin Film Gas Sensors with High Response to NH_3 in Air at Low Temperature. *Nanotechnology* **2019**, *30* (40), 405501. https://doi.org/10.1088/1361-6528/ab2d48.

(9) de Groot, F. M. F.; Grioni, M.; Fuggle, J. C.; Ghijsen, J.; Sawatzky, G. A.; Petersen, H. Oxygen 1s X-Ray-Absorption Edges of Transition-Metal Oxides. *Phys. Rev. B* **1989**, *40* (8), 5715–5723. https://doi.org/10.1103/PhysRevB.40.5715.

(10) Chaturvedi, S.; Rodriguez, J. A.; Brito, J. L. Characterization of Pure and Sulfided $NiMoO_4$ Catalysts Using Synchrotron-Based X-Ray Absorption Spectroscopy (XAS) and Temperature-Programmed Reduction (TPR). *Catal. Lett.* **1998**, *51* (1), 85–93. https://doi.org/10.1023/A:1019089002041.

(11) Chung, Y.; Lee, J. C.; Shin, H. J. Direct Observation of Interstitial Molecular N_2 in Si Oxynitrides. *Appl. Phys. Lett.* **2005**, *86* (2), 022901. https://doi.org/10.1063/1.1851620.

(12) Ohkubo, M.; Shiki, S.; Ukibe, M.; Matsubayashi, N.; Kitajima, Y.; Nagamachi, S. X-Ray Absorption near Edge Spectroscopy with a Superconducting Detector for Nitrogen Dopants in SiC. *Sci. Rep.* **2012**, *2* (1), 831. https://doi.org/10.1038/srep00831.
